# Retraction Note: TGF-β2 initiates autophagy via Smad and non-Smad pathway to promote glioma cells’ invasion

**DOI:** 10.1186/s13046-021-02219-8

**Published:** 2021-12-20

**Authors:** Chao Zhang, Xin Zhang, Ran Xu, Bin Huang, An-Jing Chen, Chao Li, Jian Wang, Xin-Gang Li

**Affiliations:** 1 Department of Neurosurgery, Qilu Hospital of Shandong University, 107, Wenhua Western Rd, Jinan, 250012 Shandong China; 2 Brain Science, Research Institute, Shandong University, 44 Wenhuaxi Road, Jinan, China; 3 Department of Biomedicine, University of Bergen, Bergen, Norway

**Retraction Note: J Exp Clin Cancer Res 36, 162 (2017)**

**https://doi.org/10.1186/s13046-017-0628-8**

The authors have retracted this article. Concerns were raised regarding a number of figures, specifically;

- Figure 1a: the panels for LC3B/WHO III and ATG5/WHO IV appear to be identical
- Figure 5b: the lanes for Smad and TFG-B2 appear to be identical
- Figure 7e: the panels for TGF-B2/LY2157299 and ATG7/LY2157299+CQ appear to be identical
- Figure 7e: the panels for ATG5/LY2157299+CQ and MMP9/LY2157299+CQ appear to be identical
- Figure 7e: the panels for ATG7/LY2157299 and MMP2/LY2157299+CQ appear to be identical
- Figure 7e: the panels for TGF-B2/Con and MMP2/LY2157299 appear to be identical

In addition, the authors have stated they found some additional errors in Figs 5c, 5d and 5j. Additionally, the authors have stated they have reviewed the original data and images that should have been included in this article, but given the extent of the misplaced figures, they have agreed to retract the article. The authors would like to apologize for any inconvenience caused by this retraction.

All authors agree to this retraction.

